# Deep Global Features for Point Cloud Alignment

**DOI:** 10.3390/s20144032

**Published:** 2020-07-20

**Authors:** Ahmed El Khazari, Yue Que, Thai Leang Sung, Hyo Jong Lee

**Affiliations:** 1 Division of Computer Science and Engineering, Jeonbuk National University, Jeonju 54896, Korea; ahmed.elghazari@gmail.com (A.E.K.); yque86@gmail.com (Y.Q.); thaileang@jbnu.ac.kr (T.L.S.); 2 Center for Advanced Image and Information Technology, Jeonbuk National University, Jeonju 54896, Korea

**Keywords:** point cloud, alignment, PointNetLK, ICP, ModelNet40

## Abstract

Point cloud registration is a key problem in computer vision applications and involves finding a rigid transform from a point cloud into another such that they align together. The iterative closest point (ICP) method is a simple and effective solution that converges to a local optimum. However, despite the fact that point cloud registration or alignment is addressed in learning-based methods, such as PointNetLK, they do not offer good generalizability for point clouds. In this stud, we proposed a learning-based approach that addressed existing problems, such as finding local optima for ICP and achieving minimum generalizability. The proposed model consisted of three main parts: an encoding network, an auxiliary module that weighed the contribution of each input point cloud, and feature alignment to achieve the final transform. The proposed architecture offered greater generalization among the categories. Experiments were performed on ModelNet40 with different configurations and the results indicated that the proposed approach significantly outperformed the state-of-the-art point cloud alignment methods.

## 1. Introduction

3D digital representations of real physical objects require a high level of data expression. 3D representations store and manipulate of information of the target objects, and they can be represented in various formats, including multi-view RGB(D), volumetric, polygonal meshes, primitive-based CAD models, or point clouds. Point clouds are collections of points in 3D space [1] that represent objects regardless of the environment. They produce fewer variations than other methods and can be observed under strong changes in lighting [2]. Several tasks can be conducted on point clouds, such as classification [3], segmentation [4], and registration [5]. However, data obtained with modern 3D sensors such as laser scanners is predominantly in the irregular format of point clouds or meshes.

Point cloud registration or alignment is a fundamental process for numerous applications including robotics [6], autonomous driving [7], augmented reality [8], and medical image processing [9]. This process shifts different sets of data into a single coordinate system [10] to match two or more images obtained using different sensors or from different viewpoints. At some point in their process, most computer vision or graphic systems require registration, such as target recognition for remote sensing, matching stereo images, and aligning medical images. In the 3D space, the problem of registration lies in finding a spatial transformation between two separate point clouds in different coordinate systems. 

The iterative closest point (ICP) [11] has been widely used to register point clouds due to its simplicity. However, it implicitly assumes that the aligned point clouds contain full overlap, which is often different from what happens in practice. Despite the existence of various techniques [12,13,14], point cloud registration has remained elusive and is an open challenge with opportunities for further improvement. Advances in data collection methods, particularly those using light detection and ranging (LiDAR) sensors [15] or portable devices such as structure sensors [16], have allowed collecting large point cloud datasets with ease. As such, most studies have adopted data-driven approaches to solve 3D-based problems. Deep learning methods presently show significant improvements in performance due to their high discriminative abilities. However, the inherent lack of structure in point clouds inhibits their use in deep learning architectures. Recent works, such as PointNet [17] and its variants [18], have made progress to overcome some of these challenges, enabling state-of-the-art methods for object detection and segmentation tasks [19,20]. Aoki et al. [21] noted that significant performance with PointNet required minimal perturbations of the point cloud in a canonical coordinate system. However, in real world scenarios, the data seldomly aligned to a canonical coordinate system. PointNetLK [21] revealed that learning-based alignment can be faster and more robust compared to classical approaches based on handcrafted features [22,23]. However, it is yet to offer better generalizability.

**Figure 1:**
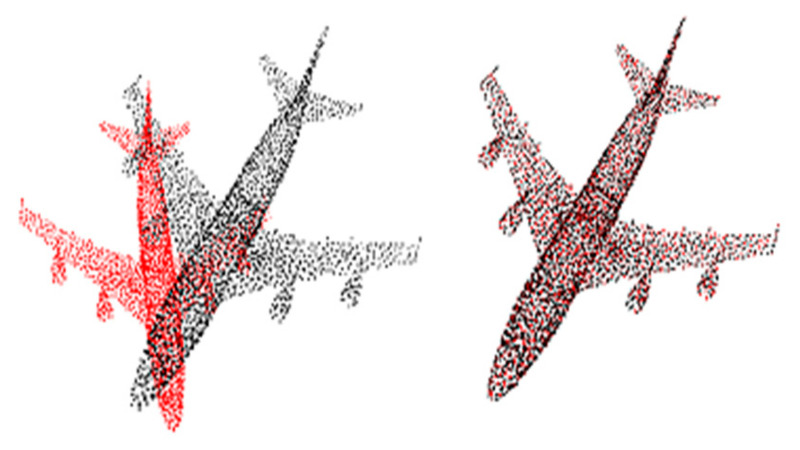
Example of desired output of point cloud registration for an airplane from ModelNet40 [24]. **Left**: before registration; **right**: after registration.

In this work, we introduce a new framework designed to solve a class of registration problems. Like ICP, this method was designed iteratively, finding a misalignment estimate. The Alignment Weight Estimation Network (Awe-Net) is a new component for our framework that includes a weight score and orientation estimator to identify the contribution of each point cloud to the final misalignment estimation using the weight scores. Furthermore, it assisted the optimization using only the 1D orientation angle. This framework provided greater generalizability and faster convergence for the correct transform estimate. This approach mainly consisted of two components: one extracted global features and the other assisted in network optimization. Compared to classical approaches based on handcrafted features to understand 3D data, the proposed approach aims to learn the global 3D features, with the model trained to estimate the transform between the target point cloud and source point cloud. Figure 1 presents an example of a desired point cloud registration output from ModelNet40 [24].

The proposed model was trained to understand the point cloud based on prior knowledge of the shape formed by the point cloud and was able to output the desired registration transform. This was achieved by robustness to noise and also by producing an estimate correct transform between the source point cloud and the required target template. Therefore, a different learning paradigm is created that consisted of extracting point cloud global features, aligning the features with the Lucas–Kanade (LK) algorithm [25], weighing the contribution of each point cloud to the final estimate using weight scores and optimizing the convergence to a correct transform by including the orientation in 1D into the loss function, resulting in improved performance. The results are then quantitatively measured utilizing ground truth and qualitatively observed. The performance of the proposed approach was evaluated based on the implicit learning of the attentive features and orientation, achieving comparable performance to state-of-the-art method on ModelNet40. 

The main contributions can be summarized as follows.

(1) A new learning paradigm was proposed for point cloud alignment, which weighed the contribution of each of the point clouds by extract global features.
(2) An Awe-Net module was proposed to obtain the estimate transform using the aligned global features. Not only does the Awe-Net output the weight scores but also the orientation of the input point cloud.
(3) The proposed model offered higher generalizability to shapes unseen during training by obtaining accurate global features.
(4) The proposed network was fully able to boost the benchmark for point alignment, exhibiting the least estimation errors among other methods. It demonstrated the ability to learn the features between the target and source point cloud by using the features produced by Awe-Net.

The rest of the paper is organized as follows: Related works are discussed in Section 2; Section 3 describes the proposed method and network architecture details; Section 4 introduces the experiments. The paper is concluded in Section 5.

## 2. Related Work 

Current registration methods can be classified into two groups: those dealing with coarse registration and those dealing with fine registration. Coarse registration methods do not consider any prior assumptions of the point cloud pose. However, fine registration or alignment algorithms assume that the input clouds are partially aligned. Hence, they use an initial proximity between the points to tweak the alignment between the two different coordinate systems.

There exists a variety of methods for local point cloud alignment, and high-end solutions are now available for applications such as SLAM [26], which requires the ICP [14] algorithm or its variants. The ICP is the most common method that iteratively performs point correspondence using the closest point and optimization using the least squares function. There are several variants of ICP [27,28] differing in their choice of cost function, how correspondences are established, and how the objective is optimized at each iteration. A noteworthy registration method that alternated with ICP is the extended Gaussian images in the Fourier domain [29], even though it required ICP to fine-tune the parameters during the final stage. However, since ICP implicitly assumes that aligned point clouds contain full overlap, ICP and its variants are generally sensitive to perturbations in alignment, producing locally optimal estimations. Since global point cloud alignment methods make no prior assumptions about the relative transformation or amount of overlap, global algorithms are often used to initialize local methods. Go-ICP [30] was developed to obtain globally optimal estimations.

There are works in literature that estimated interest points to assist with registration, i.e., scale invariant curvature descriptors [23], oriented descriptors [31], extended Gaussian images [29], fast point feature histograms [22], color intensity-based descriptors [32], global point signatures [33], heat kernels [34], and others. While interest points have the potential to improve the computational speed of the registration approaches, they are not generalized for all applications [35]. The discriminative optimization work by Vongkulbhisal et al. [36] used a hand-crafted feature vector and learned a set of maps to estimate a decent initial alignment, and the alignment was refined later using an ICP. The disadvantage of this approach was that the features and maps were specific to each object and did not generalize. More recently, they developed inverse composition discriminative optimization (ICDO) that generalized unseen object shapes. ICDO is complex, presenting the number of points in quadratic form, making it difficult to use in several real-world scenarios.

Recently, deep learning has achieved remarkable progress on point cloud registration. PointNet [17] is the representative work in the direct use of point clouds. It applies the channel-wise max pooling to aggregate per-point features into a global descriptor vector. A similar permutation equivariant layer [18] is also designed with the major difference that the permutation equivariant layer is max-normalized. Although the max-pooling idea is demonstrated to be effective, it suffers from the lack of convolutional neural networks (CNN)-like hierarchical feature aggregation. PointNet++ [37] is later proposed to group points into several groups in different levels, so that features from multiple scales could be extracted hierarchically. In the work of Ebaz et al. [38], the sub-spaces of 3D information in the form of projections or a depth map were learned using a 2D network. Two major approaches were proposed by (1) using super-points instead of key-points to find the correct transformation, and (2) encoding local 3D geometric structures using an auto-encoder. Another proposed approach was presented in [21], in which the authors proposed a modification to the Lucas–Kanade algorithm [25] to adopt it for PointNet. An extension of PointNetLK was previously presented [39], where the authors proposed a new framework that utilized a pair of MLP and a look-up table to transform point-coordinate inputs into high dimensional data. Using a deep network [40] formulates the object tracking as a relative motion estimation of two-point sets.

In this paper, we proposed a new learning approach that consisted of PointNet to extract global features and the LK algorithm was proposed in PointNetLK to align the features. Generally, this approach relies on extracted global features and the proposed Awe-Net module outputs to weigh the contribution of each point cloud to the final estimate transform during the alignment process. Our network consisted of global-features-extracting-network where we referred to PointNet and we used the LK algorithm for the point cloud alignment. As these are existing works and limited to global feature extracting, we proposed Awe-Net module outputs to weigh the contribution of each point cloud to the final estimate transform. Generally, this approach relies on extracted global features and the proposed Awe-Net module outputs to weigh the contribution of each point cloud to the final estimate transform during the alignment process. Thus, one contribution was a new Awe-Net component, which included a weight score and orientation estimator to identify the contribution of each point cloud to the final misalignment estimation using the weight scores. Predicting the orientation and the point cloud weights implicitly to the final output of the network allowed the network to converge in less iterations.

## 3. Methods

### 3.1. Features Extraction and Alignment

Global features extraction was required since we dealt with the global point cloud alignment. However, due to a lack of inherent structure of the point cloud, deep learning architectures could not directly handle the point cloud. To address this representation of the point cloud, the PointNet network was utilized as an encoding function.

ϕ denotes the PointNet function as $\phi :R^{3\times N}⟶R^{K}$, so that the input point cloud is $P\in R^{3\times N}$, $\phi \left(P\right)$ outputs a K-dimensional feature vector. The function ϕ applies to a multilayer perceptron (MLP) for each 3D point in P. A symmetric pooling function is then applied, followed by a returning K-dimensional global descriptor.

Aoki et al. [21] proposed a modification of the LK algorithm to handle the feature alignment. Thus, let $\xi_{iT_{i}}\in se\left(3\right)$ and $P_{T},P_{S}$ be the template and source point clouds, respectively, with an aim of finding the rigid-body transform $G\in SE\left(3\right),$ which aligns the source to the template.

(1) $$G=\exp\left(\underset{i}{\sum }\xi_{i}T_{i}\right)\;\xi =\left(\xi_{1},\xi_{2},\ldots ,\xi_{6}\right),$$

where $T_{i}$ are the generators of the exponential map with twist parameters $\xi \in R^{𝟞}$. Now, the problem of alignment is defined to find *G* such that $\phi \left(P_{T}\right)=\phi \left(G\cdot P_{S}\right)$. To achieve that, an iterative optimization solution is used with regards to inverse compositional (IC) formulation [41].

(2) $$\phi \left(P_{S}\right)=\phi \left(G^{-1}\cdot P_{T}\right)$$

(3) $$\phi \left(P_{S}\right)=\phi \left(P_{T}\right)+\frac{\partial }{\partial \xi }\left[\phi \left(G^{-1}\cdot P_{T}\right)\right]\xi$$

The Jacobian is denoted as $J=\frac{\partial }{\partial \xi }\left[\phi \left(G^{-1}\cdot P_{T}\right)\right],$

where $J\in R^{K\times 6}$ is the matrix. Using a stochastic gradient approach, J becomes:

(4) $$J_{i}=\frac{\phi \left(\exp\left(-t_{iT_{i}}\right)\cdot P_{T}\right)-\phi \left(P_{T}\right)}{t_{i}}$$

Equation (3) becomes:

(5) $$\xi =J^{+}\left[\phi \left(P_{S}\right)-\phi \left(P_{T}\right)\right],$$

where $J^{+}$ is the Moore–Penrose inverse of J.

### 3.2. Alignment Weight Estimation Network

The Awe-Net architecture module was proposed to obtain the estimate transform using the procedure described in the feature’s extraction and alignment procedure. This module was the MLP-based network that learned important weights to evaluate the contribution of each input, which regularized ξ, as described in Equation (5). Not only did the Awe-Net output the weight scores but also the orientation of the input point cloud.

**Figure 2:**
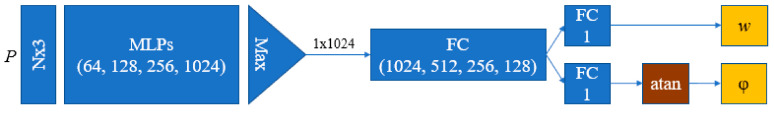
Awe-Net architecture: The model consists of four multi-layer perceptions (MLPs) with sizes of 64, 128, 256, and 1024. Using a max-pooling function allows the model to obtain auxiliary features that are followed by four fully connected layers (1024, 512, 256, and 128) that later branch out into the orientation and weight score.

A block diagram of the architecture is presented as in Figure 2. The model consisted of three MLP with sizes of 64, 128, 256, and 1024. Then a symmetric max-pooling function was used to find the auxiliary features. Later, these features were assigned to several fully connected layers. In this work, four fully connected layers were selected, as they proved empirically sufficient for robust performance. The FC layers consisted of the following nodes: 1024, 512, 256, and 128. Later, the last FC layer branched into 1-layer paths for orientation and weight score predictions. The weight score, W, was a positive number that indicated the most prominent features. This score was learned in different positions to the input point cloud since learning was processed iteratively.

We only predicted a 1D rotation angle φ, avoiding equivariances in order to retain a higher discriminating power. This was reasonable since the role of Awe-Net is to assist training to converge to the best possible parameters during training, whereas the overall transform is predicted in a different stream.

### 3.3. Proposed Architecture

**Figure 3:**
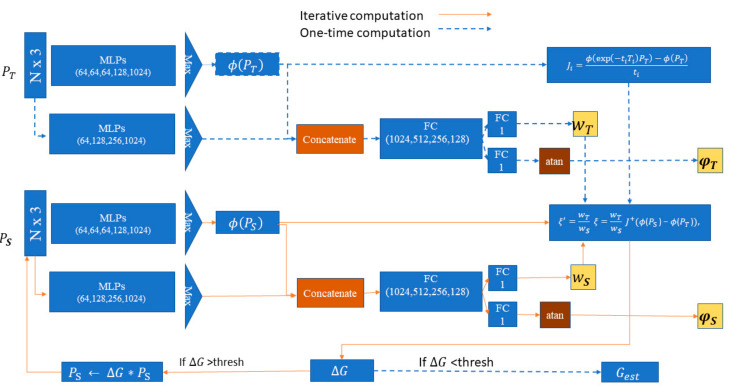
Point cloud source P_S_ and template P_T_ are fed into shared MLP, and max-pooling to obtain the global features ϕ (P_S_) and ϕ (P_T_) then the Jacobian J is computed using ϕ(P_T_). Both input clouds are passed through Awe-Net to return the weight scores W_S_·W_T_ and the orientations *φ_S_* and *φ_T_*. The optimal modified twist parameters $\xi {｜}^{\prime }$ are obtained and used to iteratively update the pose of P_S_, and then the global feature vector $\phi \left(P_{S}\right)$ is recalculated. During training, the loss function is based on the difference between the orientations $\varphi_{S}$ and $\varphi_{T}$ and between the corresponding points in source and template point cloud.

This section introduces our proposed architecture. A diagram of the architecture is presented in Figure 3. The model consisted of five MLPs similar to PointNet with sizes of 64, 64, 128, and 1024. Both source $P_{S}$ and template $P_{T}$ were assigned to the MLP in a Siamese architecture [42] followed by max pooling as a symmetric function, resulting in global feature vectors $\phi \left(P_{S}\right)$ and $\phi \left(P_{T}\right)$.

In Figure 3, one can observe another stream going through the Awe-Net to obtain the scores W and the orientation ϕ. The features extracted in the first and second streams were later concatenated before feeding them to the FC layers. The reason behind the addition of the concatenated features was to provide the Awe-Net with all relevant features, such that the overall network converged. The scores of both $P_{S}$ and $P_{T}$ needed to have similar contributions in the transform estimation to optimize the twist parameters. Therefore, Equation (5) became:

(6) $$\xi {｜}^{\prime }=\frac{W_{T}}{W_{S}}\xi =\frac{W_{T}}{W_{S}}J^{+}\left[\phi \left(P_{S}\right)-\phi \left(P_{T}\right)\right],$$

where $W_{T}$ and $W_{S}$ are the weight scores of the template and the source point clouds, respectively.

Meanwhile, the orientations of the source *φ*_S_ and the template *φ*_T_ should both be equal, which means that for each iteration, the orientation *φ_S_* tends to converge to *φ_T_*.

The network was presented through an iterative scheme. In addition, introducing the iterations empirically allowed the use of lesser hidden layers in Awe-Net. Using the proposed loss function, the model could achieve comparable results with less parameters, since the iterations guarantee continuous update of the parameters of the model instead of one-shot model which will require more distinctive features. The model was not pretrained on the classification that helped distinguish among categories, requiring more iterations to converge.

The algorithm consisted of a looping computation of the optimized twist parameters (6).

(7) $$P_{S}⟵\Delta G\cdot P_{S,}\;\mathrm{where}\;\Delta G=\exp\left(\sum_{i}\xi_{i}^{{｜}^{\prime }}{\;T}_{i}\right)$$

After performing n iterations, the overall transformation between the original source and template point clouds was obtained by combining all transforms in each iteration:

(8) $$G_{\mathrm{est}}={\Delta G}_{n}\cdot \cdot \cdot \cdot {\Delta G}_{0}$$

The convergence criteria were also used

(9) ΔG<ε,

where ε represents the stopping criterion for iterations. We used $\varepsilon =10^{7}$.

### 3.4. Loss Function

The objective of the loss function is to minimize the distance between the corresponding points in the source and template point cloud as well as the error between $\varphi_{S}$ and $\varphi_{T}$. The earth mover distance (EMD) [43] was used to compute the difference between the source point cloud and the template, although there were other alternatives such as PoseLoss [44] or the L2 between the estimated transform matrix. EMD showed its effectiveness in learning using the iterative approach [45]. The L1 distance was used to compute the orientation loss. The overall loss is given by:

(10) $$L=\underset{\Psi :P_{S}^{est}\to P_{T}}{min}\frac{1}{\left|P_{S}^{\mathrm{est}}\right|}\underset{x\in \;P_{S}^{est}}{\sum }∥x-\Psi \left(x\right){∥}_{2}+\left|\phi_{S}-\phi_{T}\right|,$$

where $P_{T}$ is the template point cloud, $P_{S}$ is the source point cloud, and $P_{S}^{est}$ represents the transformed source point cloud by the estimated transformation G from (1). The EMD finds a bijection ψ and minimizes the distance between corresponding points based on ψ.

## 4. Experiments and Results

### 4.1. Dataset

**Figure 4:**
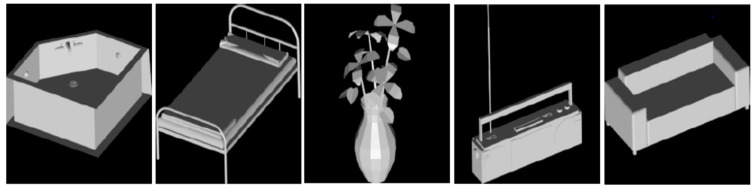
Illustration of models in ModelNet40 (bathtub, bed, flowerpot, radio, and sofa).

In this section, we introduce the dataset of 3D models used for training and testing. ModelNet [24] is one of the most recognized and commonly used datasets containing 3D models in a mesh format. It was developed at Princeton University. Its subset, ModelNet40, is used as a benchmark for testing different approaches. This dataset is used as the main focus for the evaluations. ModelNet40 contains 40 different categories and 12,311 individual models. The dataset has an official split for training and testing subsets. Figure 4 shows examples of models in ModelNet40.

The models in the original ModelNet40 are not aligned and have widely different scales. Therefore, when processing the data for neural networks, all models require to be scaled to fit a unit sphere. The categories in the dataset are not equally populated. For instance, there are over 700 airplane models and only over 100 wardrobe models. ModelNet40 contains files in the off format. Therefore, preprocessing is required to generate the point clouds from the meshes and sub-sample them to feed the neural network.

### 4.2. Experimental Results

In this paper, various training combinations were performed using the training data and the test data. The results were then compared to those of PointNetLK as the baseline and with the ICP algorithm. The networks were trained in three different scenarios: (1) Training and testing on fully visible data, (2) training and testing on partially visible data, and (3) training and testing with additive noise. For the proposed method and other PointNetLK-based approaches, a maximum of 20 iterations were allowed when performing tests, while the maximum iterations for ICP were 100. 

#### 4.2.1. Train and Test on Fully Visible Data

**Experiment 1:** In the first experiment, the proposed model and PointNetLK were trained on 20,175 different data in ModelNet40, then tested on the test set for the same 20 categories. There was no noise in the source data during training and testing.

**Figure 5:**
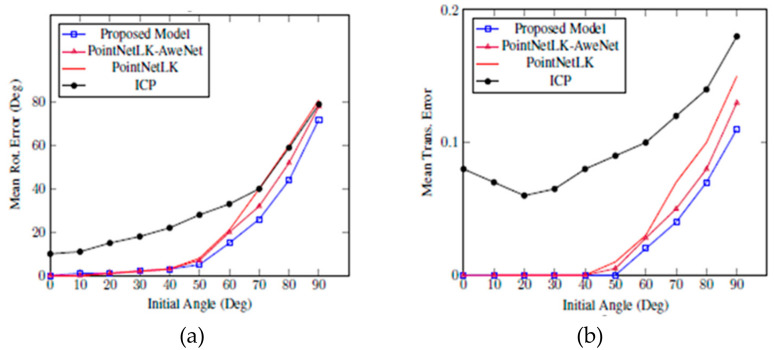
Our proposed model trained on 20,175 different data in ModelNet40. The model was tested using Mean Rot. Error (**a**) and Mean Trans. Error (**b**). Both figures show that our proposed model outperforms iterative closest point (ICP) and PointNetLK in alignment on categories seen during training.

The standard version of PointNet for classification was trained first using ModelNet40. Therefore, the feature extractor ϕ of the proposed model was initialized using the trained PointNet model and then fine-tuned with the proposed loss function in (3.9). $G_{\mathrm{est}}$ was used in the training with random rotation angles of 0, 45 degrees and translations of 0, 0.8 in three-axes. During the test phase, the initial translations were within 0, 0.3 and initial rotations within 0, 90 degrees. Figure 5 presents the results after 10 iterations of ICP, PointNetLK, PointNetLK-AweNet, and the proposed model. This indicates that the proposed model allows faster convergence to the correct solution with fewer iterations.

**Figure 6:**
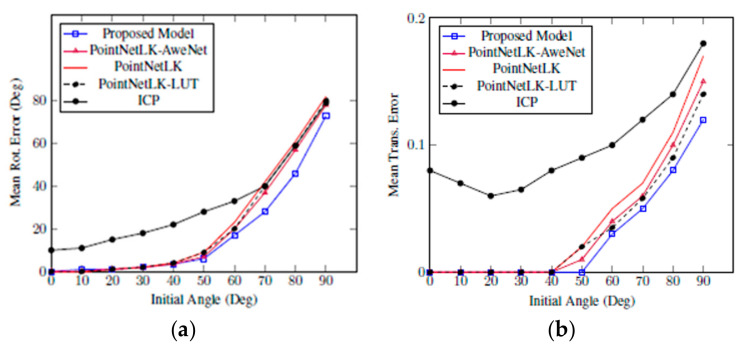
Our proposed model trained on the other 20 categories of ModelNet40. The model was tested using Mean Rot. Error (**a**) and Mean Trans. Error (**b**). Both figures show that our proposed model outperforms ICP and PointNetLK-based approaches in alignment on categories unseen during training.

**Experiment 2:** To test the generalizability of the proposed model, we repeated experiment 1, yet the training in this experiment was done on the other 20 categories of ModelNet40. The test was performed on 20 categories in ModelNet40, which were in the same categories as Experiment 1. The proposed model was able to generalize much more precisely than PointNetLK and ICP for alignment on object categories that were not seen in the training. Figure 6 shows the results obtained for ModelNet40 through Experiment 2. In both experiments, the results for 10 iterations are reported, showing the proposed model performs a correct alignment in fewer iterations.

Our method in Experiment 1 and Experiment 2 produced better results compared to others. Mean rotational error at the left graph shows that our proposed model obtained less errors compared to the existing state-of-the-art methods, such as ICP, PointNetLK, and PointNetLK-LUT. Mean translational error at the right graph also exhibits the comparison results with ICP, PointNetLK, and PointNetLK-LUT, obtaining less error. Moreover, when we applied Awe-Net to PointNetLK, the errors were greatly reduced.

In both experiments, all approaches produced lower errors from angle 0 to 50 degrees, but the errors rose rapidly afterward. However, our approach remained the lowest one. Our model trained only 10 iterations, yet it was sufficient due to its consistent results in every angle of the range from 0 to 90 degrees.

#### 4.2.2. Train and Test on Partially Visible Data

In a real-world scenario, the template used is commonly a 3D model and the sources are taken from the scanner. To reproduce the same environment in the real world, sampling visible points is typically based on simulating a 3D sensor that has a horizontal and vertical field-of-view and a minimum and maximum depth. The sampling is done on the ModelNet40 dataset following the same process conducted in the PointNetLK. The faces of the template are sampled and placed into a unit box ${\left[0,1\right]}^{3}$, the source was then warped using some random perturbation. The template and the source were translated by $2\cdot {\left[1,1,1\right]}^{T}$ from the origin. Thus, let $P_{T}^{v}\;$ denote all the points that satisfy $\left(P_{T}+2\cdot {\left[1,1,1\right]}^{T}\right)<\mathrm{mean}\left(P_{T}+2\cdot {\left[1,1,1\right]}^{T}\right)$, which means placing the sensor in the direction of ${\left[1,1,1\right]}^{T}$ and sampling the point cloud in front of the sensor—in other words, the visible part of the 3D object. 

**Figure 7:**
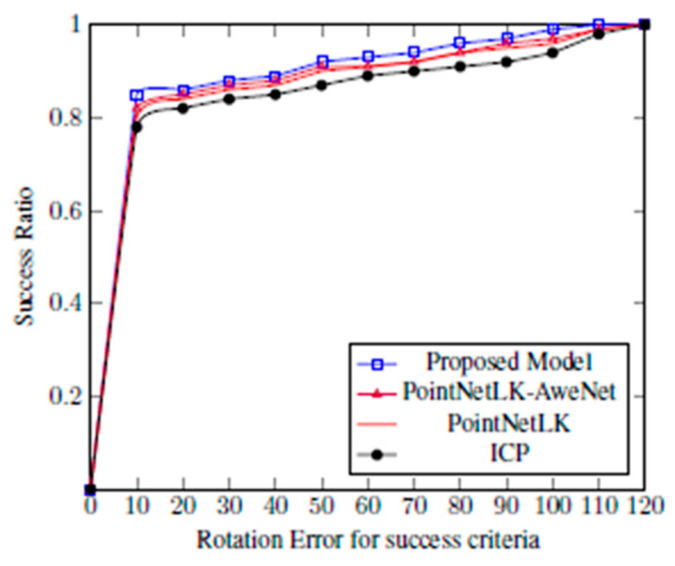
The alignment of the data that are partially visible is tested on ModelNet40.

**Figure 8:**
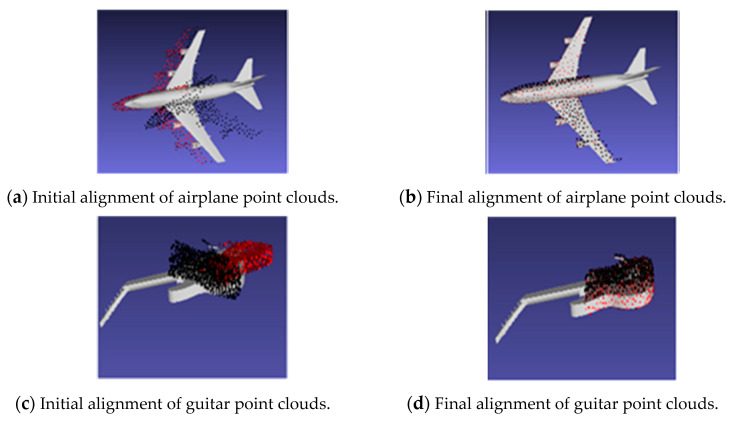
Illustration of point alignment of partially visible data. The alignment test on partially visible data was conduced on ModelNet40. The results include PointNetLK shown by black points. The proposed method is shown by red points. (**a**) initial alignment of airplane point clouds; (**b**) final alignment of airplane point clouds; (**c**) initial alignment of guitar point clouds; (**d**) final alignment of guitar point clouds.

**Figure 9:**
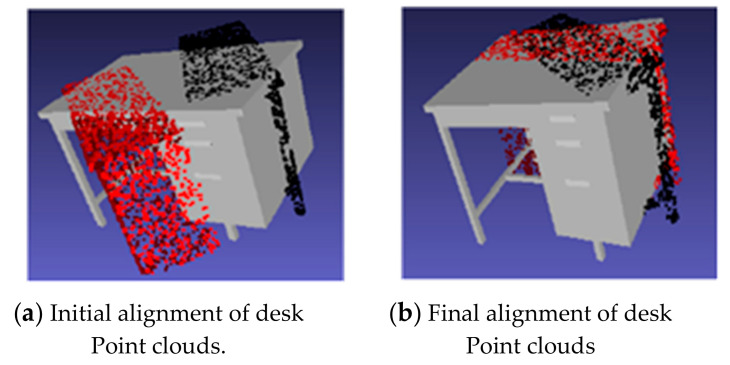
Illustration of failure case point alignment of partially visible data. The alignment test on partially visible data of ModelNet40. The results include PointNetLK shown by black points and the proposed method shown by red points.

The test on the ModelNet40 was conducted using random translation between 0, 0.3. The area under the curve (AUC) was used as the metric to evaluate the performance of the registration algorithms. Plots showing the success ratio versus success criteria on rotation error (in degrees) were generated for ICP, PointNetLK, PointNetLK-AweNet, and the proposed model. Figure 7 shows examples of these curves. The area below the curves was divided by 120 to normalize between 0 and 1, and it was defined as AUC. The y-axis was the successful ratio of experiments and the x-axis shows rotation error for success criteria, which was set to determine the alignment estimation success. Thus, the x-axis showed a maximum rotation error value that qualified the estimation to be a success. Therefore, the area under the curve (or the integral not equal to 1 in this case) divided the 120 maximum degree error of the x-axis to make the AUC<= 1. AUC expressed a measure of registration success and, as such, the higher the value of AUC, the better the network’s performance. The proposed model was able to generalize well and register objects using the sensor model approach. When the rotation error was less than 5 degrees and the translation less than 0.01, it was considered a success. Figure 8 shows examples of the template and partially visible source point clouds that were aligned for the ModelNet test dataset. The success of the qualitative results was due to the fact that the model offered good generalizability. Conversely, Figure 9 shows a case of failure regarding alignment due to the symmetry of a point cloud. The proposed model was not trained to extract symmetric information of the 3D model. In Figure 9, the table is symmetric based on a certain plane where misalignment happens. Moreover, the proposed model did not consider the geometric information of the point cloud since the feature extractor was PointNet.

#### 4.2.3. Train and Test with Additive Noise

**Figure 10:**
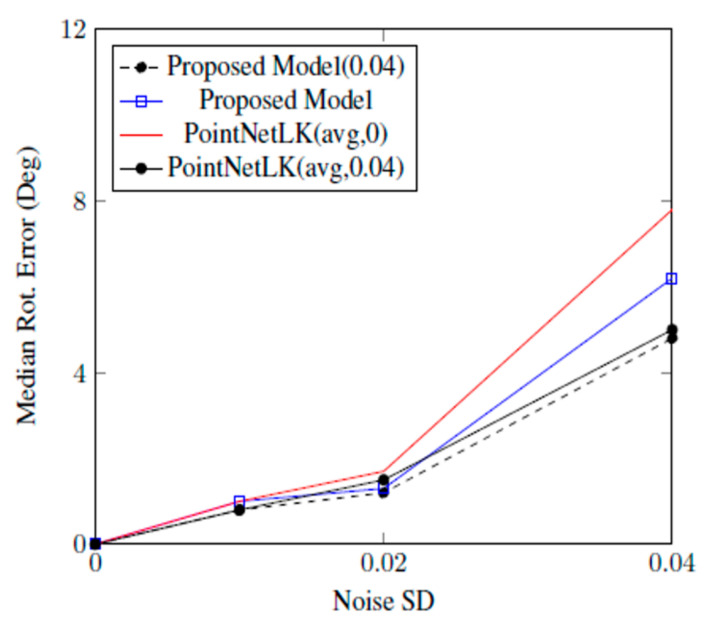
The proposed model outperforms ICP and PointNetLK in alignment on noisy data.

This section presented experiments conducted with additive Gaussian noise. The template point cloud was randomly sampled from faces and the source point cloud was equal to the template with additive Gaussian noise at a certain standard deviation. The first 1000 points were selected during the sampling process. The authors of PointNetLK hypothesized that the choice of the symmetric operator was critical to the performance with additive noise. PointNet used a max pool operator yield to output the global feature vector. In case of noisy data, the choice was subject to variations among the different random noise samples. Therefore, PointNetLK used average pooling when learning global features rather than the max pool operator, assuming that it would be better suited to learn global features. However, the proposed model was trained using the same symmetric operator, which meant that the max-pooling operator was used during training. Figure 10 shows the results of the proposed model with PointNetLK. The proposed model was trained on zero-noise data and then trained on noisy data with a standard deviation SD = 0.04. The proposed model achieved better performance than PointNetLK, with an average pooling and training noise of SD = 0.04. 

The shortcoming of approaches based on hand-crafted features [35,36] (learned alignment) was a quadratic complexity in the number of points and a lack of generalization due to the feature vector and registration maps both being learned. Moreover, we found that the PointNetLK approach produced good generalizability to shapes unseen in training but was not robust to noise [21]. In Figure 8, Figure 9 and Figure 10, we quantitively proved that our approach outperformed the state-of-the-art alignment approach in various scenarios, meaning that our model provided better generalizability.

## 5. Conclusions

We proposed a novel learning approach to solve the point cloud alignment problem with highly accurate registration, based on an effective auxiliary Awe-Net module that assisted with the overall network in converging to the correct estimate and learning in order to apply the contribution of each input point clouds. The proposed network was implemented in an iterative manner to obtain highly accurate transform estimates comparable to other global registration methods. The advantage of our framework was that it provided a good approach for different scenarios where prior knowledge existed of the shape formed by the point cloud and where noise was present. The Awe-Net showed its robustness to noise and initial random misalignment. It provided higher generalizability to shapes unseen during training and was fully able to boost the benchmark for point alignment. Experimental results showed the robustness of the proposed framework to noisy real-world data, partial overlap data, and fully visible data. Despite the success of this approach, there is still room for improvement since the network can only be implemented iteratively. We aim to adopt a single-shot design as well as handling occluded point clouds. 

## References

[1] Robert A., Vijay K. (2008). Robotics: State of the Art and Future Challenges.

[2] Yew Z.J., Lee G.H. (2018). 3DFeat-Net: Weakly Supervised local 3D features for point cloud registration. European Conference on Computer Vision.

[3] Munoz D., Vandapel N., Hebert M. Directional Associative Markov Network for 3-d Point Cloud Classification. https://kilthub.cmu.edu/articles/Directional_Associative_Markov_Network_for_3-D_Point_Cloud_Classification/6552644/1.

[4] Nguyen A., Le B. 3D point cloud segmentation: A survey. Proceedings of the 2013 6th IEEE Conference on Robotics, Automation and Mechatronics (RAM).

[5] Tam G.K.L., Cheng Z.-Q., Lai Y.-K., Langbein F.C., Liu Y., Marshall D., Martin R.R., Sun X.-F., Rosin P.L. (2012). Registration of 3D point clouds and meshes: A survey from rigid to nonrigid. IEEE Trans. Vis. Comput. Graph..

[6] Jerbić B., Šuligoj F., Švaco M., Šekoranja B. (2015). Robot assisted 3D point cloud object registration. Procedia Eng..

[7] Chen X., Ma H., Wan J., Li B., Xia T. Multi-view 3d object detection network for autonomous driving. Proceedings of the 2017 IEEE Conference on Computer Vision and Pattern Recognition.

[8] Alexiou E., Upenik E., Ebrahimi T. Towards subjective quality assessment of point cloud imaging in augmented reality. Proceedings of the 2017 IEEE 19th International Workshop on Multimedia Signal Processing (MMSP).

[9] Sitek A., Huesman R.H., Gullberg G.T. (2006). Tomographic reconstruction using an adaptive tetrahedral mesh defined by a point cloud. IEEE Trans. Med Imaging.

[10] Brown L.G. (1992). A survey of image registration techniques. Acm Comput. Surv. Csur.

[11] Besl P.J., McKay N.D. (1992). Method for registration of 3-D shapes. Proceedings Volume 1611, Sensor Fusion IV: Control Paradigms and Data Structures.

[12] Serafin J., Grisetti G. NICP: Dense normal based point cloud registration. Proceedings of the 2015 IEEE/RSJ International Conference on Intelligent Robots and Systems (IROS).

[13] Men H., Gebre B., Pochiraju K. Color point cloud registration with 4D ICP algorithm. Proceedings of the 2011 IEEE International Conference on Robotics and Automation.

[14] Mitra N.J., Gelfand N., Pottmann H., Guibas L. Registration of Point Cloud Data from a Geometric Optimization Perspective. https://dl.acm.org/doi/abs/10.1145/1057432.1057435.

[15] Lidar—Light Detection and Ranging—Is a Remote Sensing Method Used to Examine the Surface of the Earth. https://oceanservice.noaa.gov/facts/lidar.html.

[16] Structure Sensor. https://structure.io/.

[17] Qi C.R., Su H., Mo K., Guibas L.J. Pointnet: Deep learning on point sets for 3d classification and segmentation. Proceedings of the IEEE Conference on Computer Vision and Pattern Recognition.

[18] Ravanbakhsh S., Schneider J., Poczos B. (2016). Deep learning with sets and point clouds. arXiv.

[19] Qi C.R., Liu W., Wu C., Su H., Guibas L.J. Frustum Pointnets for 3d Object Detection from rgb-d Data. https://openaccess.thecvf.com/content_cvpr_2018/papers/Qi_Frustum_PointNets_for_CVPR_2018_paper.pdf.

[20] Yuan W., Held D., Mertz C., Hebert M. (2018). Iterative Transformer Network for 3D Point Cloud. arXiv.

[21] Aoki Y., Goforth H., Srivatsan R.A., Lucey S. Pointnetlk: Robust & efficient point cloud registration using pointnet. Proceedings of the IEEE Conference on Computer Vision and Pattern Recognition.

[22] Rusu R.B., Blodow N., Beetz M. Fast point feature histograms (FPFH) for 3D registration. Proceedings of the 2009 IEEE International Conference on Robotics and Automation.

[23] Gelfand N., Mitra J., Guibas L.J., Pottmann H. Robust Global Registration. https://www.dmg.tuwien.ac.at/geom/ig/publications/oldpub/2005/gmgp_registration_05/paper_docs/registration.pdf.

[24] Wu Z., Song S., Khosla A., Yu F., Zhang L., Tang X., Xiao J. 3d Shapenets: A Deep Representation for Volumetric Shapes. https://people.csail.mit.edu/khosla/papers/cvpr2015_wu.pdf.

[25] Lucas B.D., Kanade T. An Iterative Image Registration Technique with an Application to Stereo Vision. https://www-pequan.lip6.fr/~bereziat/cours/master/vision/papers/lucas81.pdf.

[26] Durrant-Whyte H., Bailey T. (2006). Simultaneous localization and mapping: Part I. IEEE Robot. Autom. Mag..

[27] Rusinkiewicz S., Levoy M. Efficient variants of the ICP algorithm. Proceedings of the third International Conference on 3-D Digital Imaging and Modeling.

[28] Arun Srivatsan R., Xu M., Zevallos N., Choset H. (2018). Probabilistic pose estimation using a Bingham distribution-based linear filter. Int. J. Robot. Res..

[29] Makadia A., Patterson A., Daniilidis K. Fully automatic registration of 3D point clouds. Proceedings of the 2006 IEEE Computer Society Conference on Computer Vision and Pattern Recognition.

[30] Yang J., Li H., Jia Y. Go-icp: Solving 3d registration efficiently and globally optimally. Proceedings of the IEEE International Conference on Computer Vision.

[31] Glover J., Bradski G., Rusu R.B. (2012). Monte carlo pose estimation with quaternion kernels and the bingham distribution. Robotics: Science and Systems.

[32] Godin G., Rioux M., Baribeau R. (1994). Three-dimensional registration using range and intensity information. Videometrics III.

[33] Chua C.S., Jarvis R. (1997). Point signatures: A new representation for 3d object recognition, Point signatures: A new representation for 3d object recognition. Int. J. Comput. Vis..

[34] Ovsjanikov M., Mérigot Q., Mémoli F., Guibas L. (2010). One point isometric matching with the heat kernel. Computer Graphics Forum.

[35] Guo Y., Bennamoun M., Sohel F., Lu M., Wan J. (2014). 3D object recognition in cluttered scenes with local surface features: A survey. IEEE Trans. Pattern Anal. Mach. Intell..

[36] Vongkulbhisal J., De la Torre F., Costeira J.P. Discriminative Optimization: Theory and Applications to Point Cloud Registration. https://openaccess.thecvf.com/content_cvpr_2017/papers/Vongkulbhisal_Discriminative_Optimization_Theory_CVPR_2017_paper.pdf.

[37] Qi C.R., Yi L., Su H., Guibas L.J. Pointnet++: Deep Hierarchical Feature Learning on Point Sets in a Metric Space. https://papers.nips.cc/paper/7095-pointnet-deep-hierarchical-feature-learning-on-point-sets-in-a-metric-space.pdf.

[38] Elbaz G., Avraham T., Fischer A. 3D point cloud registration for localization using a deep neural network auto-encoder. Proceedings of the IEEE Conference on Computer Vision and Pattern Recognition.

[39] Sekikawa Y.S.T. (2019). Tabulated MLP for Fast Point Feature Embedding. arXiv.

[40] Groß J., Ošep A., Leibe B. Alignnet-3d: Fast point cloud registration of partially observed objects. Proceedings of the 2019 International Conference on 3D Vision (3DV).

[41] Baker S., Matthews I. (2004). Lucas-kanade 20 years on: A unifying framework. Int. J. Comput. Vis..

[42] Chopra S., Hadsell R., LeCun Y. Learning a similarity metric discriminatively, with application to face verification. Proceedings of the 2005 IEEE Computer Society Conference on Computer Vision and Pattern Recognition.

[43] Rubner Y., Tomasi C., Guibas L.J. (2000). The earth mover’s distance as a metric for image retrieval. Int. J. Comput. Vis..

[44] Xiang Y., Schmidt T., Narayanan V., Fox D. (2017). Posecnn: A convolutional neural network for 6d object pose estimation in cluttered scenes. arXiv.

[45] Fan H., Su H., Guibas L.J. A Point Set Generation Network for 3d Object Reconstruction from A Single IImage. https://ai.stanford.edu/~haosu/papers/SI2PC_arxiv_submit.pdf.

